# Chemigenetic Encoding of Fluorescent Dyes Enables High‐Fidelity and Wash‐Free Imaging of Proteins in Live Cells

**DOI:** 10.1002/advs.202505967

**Published:** 2025-06-20

**Authors:** Xuelian Zhou, Lu Miao, Ning Xu, Yonghui Chen, Jinjing Shi, Jinhua Zhou, Guangying Wang, Qinglong Qiao, Zhaochao Xu

**Affiliations:** ^1^ Dalian Institute of Chemical Physics Chinese Academy of Sciences 457 Zhongshan Road Dalian 116023 China; ^2^ School of Chemistry Dalian University of Technology 2 Linggong Road Dalian 116024 China

**Keywords:** dyes, fluorescent proteins (FPs), förster resonance energy transfer (FRET), rhodamine, structured illumination microscopy (SIM)

## Abstract

Fluorescent dyes are indispensable chemical tools for protein labeling, yet their utility in live‐cell imaging remains constrained by background fluorescence from off‐target interactions. A chemigenetic strategy is presented that integrates synthetic dye chemistry with genetically encoded fluorescence to achieve high‐fidelity, wash‐free imaging of target proteins. By leveraging Förster resonance energy transfer (FRET) between fluorescent proteins (FPs) and Halo‐tag dyes, a system is engineered where fluorescence emission depends on FPs donor excitation, ensuring only dyes bound to target protein are fluorescent, while nonspecifically bound dyes remain dark, enhancing the signal‐to‐noise ratio (SNR). This approach is implemented by fusing Halo‐tag with FPs (sfGFP, mCherry) to create FRET pairs with Halo dyes (**O‐Rho**, **Si‐Rho**), yielding chemigenetic fluorophores (GLH‐O, CLH‐Si) with improved SNR even at low expression levels. By optimizing FRET efficiency, the developed probes CH‐Si are combined with standard FPs and commercial dyes, and achieve four‐color structured illumination microscopy (SIM) imaging of target proteins, and track the mitochondria interactions with endoplasmic reticulum, and lysosomes. Furthermore, by incorporating the ultra‐stable mStayGold as donor, a photostable fluorophore (SLH‐O) capable of long‐term super‐resolution imaging is developed. This versatile strategy combines chemical and genetic tools, offering a generalizable platform for high‐precision studies of protein and cellular processes.

## Graphical abstract

This study develops a chemigenetic strategy that combines synthetic dyes with genetically encoded fluorescence for high‐fidelity, no‐wash protein imaging. By utilizing FRET between fluorescent proteins and Halo‐tag dyes, this system ensures that only target‐bound dyes fluoresce, effectively suppressing background signals and enhancing the signal‐to‐noise ratio.

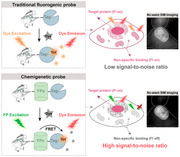

## 1 Introduction

The ability to visualize and manipulate proteins in living cells has been transformative for modern biology, enabling researchers to dissect complex cellular processes with spatiotemporal precision.^[^ ^1^, ^2^ ^]^ Central to this progress are small‐molecule fluorescent dyes, which have become indispensable chemical tools in chemical biology due to their exceptional brightness, photostability, and versatility.^[^ ^3^, ^4^, ^5^ ^]^ When paired with self‐labeling protein tags such as Halo‐tag and SNAP‐tag, these dyes enable specific labeling of target proteins in live cells.^[^ ^6^, ^7^, ^8^ ^]^ However, the use of exogenous dyes is often hampered by nonspecific binding to off‐target cellular components, leading to significant background fluorescence that degrades imaging quality.^[^ ^9^, ^10^ ^]^ Even extensive washing protocols cannot fully eliminate these artifacts, posing a major challenge for quantitative imaging of low‐abundance proteins and single‐molecule tracking.^[^ ^11^, ^12^, ^13^ ^]^

**Scheme 1:**
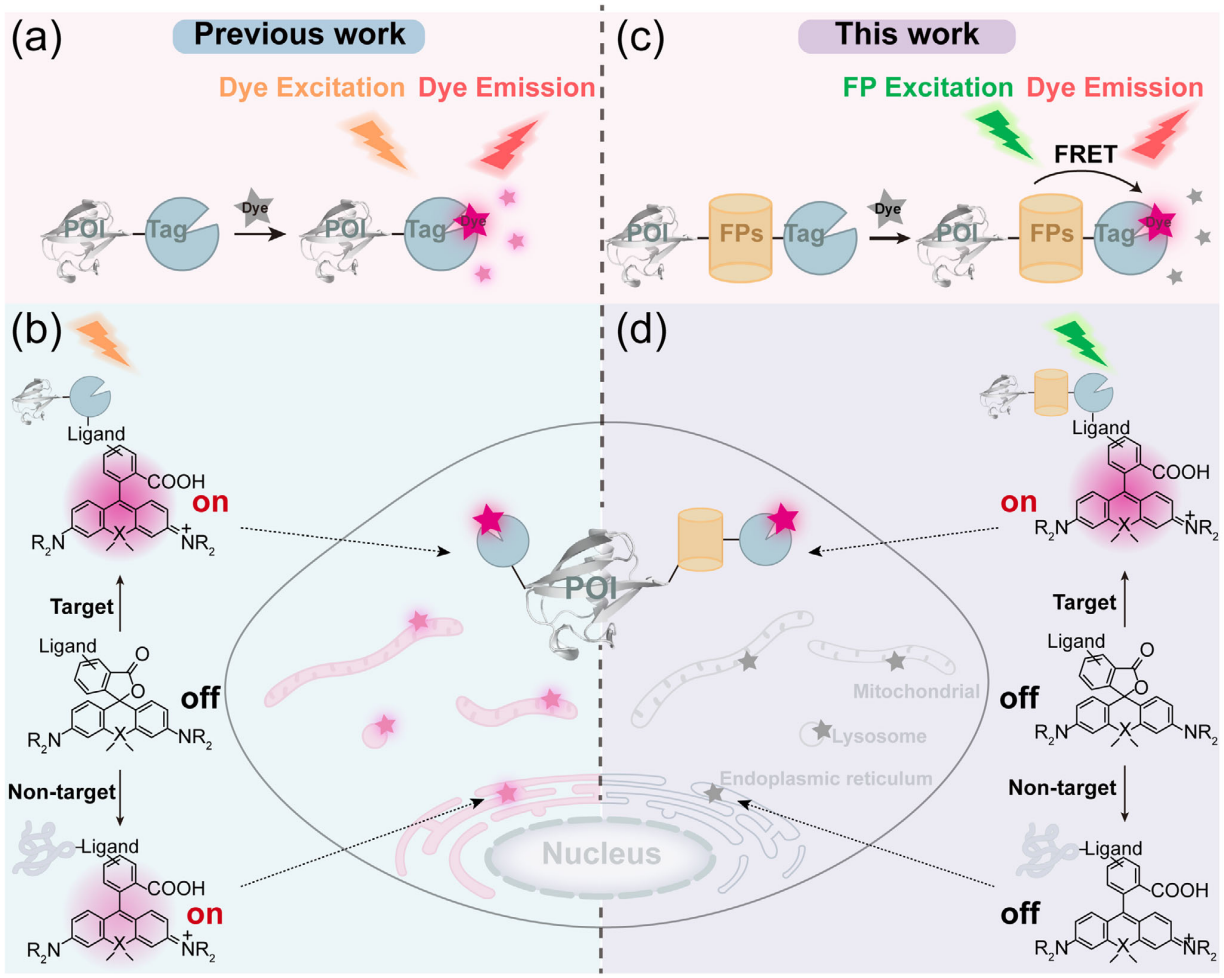
Comparison of fluorogenic probe design strategies based on protein tags and FRET. a) Traditional fluorogenic probes of self‐labeling protein tags. b) The environment‐dependent switch between the spirolactones (L) and zwitterions (Z) in Rhodamine is prone to nonspecific background signals. c) The genetically coded fluorophores were constructed by fusing the Halo‐tag to fluorescent proteins. d) It avoids direct excitation of non‐specifically dye fluorescence and effectively reduce background fluorescence.

To address these limitations, chemical biologists have developed “fluorogenic” dyes that combine genetic encoding with in situ fluorescence activation, effectively mimicking the behavior of fluorescent proteins.^[^ ^14^, ^15^ ^]^ For example, protein tag‐based fluorogenic dyes have been designed to activate fluorescence upon binding to their cognate tags. However, these dyes are not entirely specific, as interactions with off‐target cellular components can also induce fluorescence (**Scheme** **1**a,b).^[^ ^16^, ^17^, ^18^ ^]^ A well‐studied example is the rhodamine‐based probes, whose fluorescence is governed by the equilibrium between non‐fluorescent spirolactones (L) and fluorescent zwitterions (Z).^[^ ^19^, ^20^ ^]^ While binding to the target tag shifts this equilibrium toward the fluorescent Z state, intracellular factors such as pH,^[^ ^21^ ^]^ polarity,^[^ ^22^ ^]^ metal ions,^[^ ^23^ ^]^ and hydrophobic protein cavities^[^ ^24^ ^]^ can also trigger this conversion, leading to unwanted background signals, such as nonspecific binding to mitochondria or lysosomes (Scheme 1b).^[^ ^25^, ^26^ ^]^ Recently, typical fluorogenic probes have been developed using bioorthogonal reactions to enable the in situ generation of fluorophores with large Stokes shifts within cells,^[^ ^27^ ^]^ yet achieving universal fluorogenicity for any dye during protein recognition remains a significant challenge.

Here, we introduce a chemigenetic strategy that bridges the gap between synthetic dye chemistry and genetically encoded fluorescence, offering a robust solution to the problem of background fluorescence in cell imaging. Our approach leverages Förster resonance energy transfer (FRET) between a fluorescent protein (FP) donor and a dye acceptor,^[^ ^28^, ^29^ ^]^ thereby conferring genetically encoded fluorescence properties to the dye (λ_ex_ = FP excitation wavelength, λ_em_ = dye emission wavelength) (Scheme 1c). In previous studies, this hybrid FRET strategy had been used to enhance the optical stability of fluorescent proteins.^[^ ^30^, ^31^ ^]^ By ensuring that only dye molecules bound to the target protein are excited via FRET, we minimize background fluorescence from nonspecifically bound dyes. This strategy not only improves imaging fidelity but also eliminates the need for extensive washing steps, making it ideal for dynamic and long‐term imaging applications.

Specifically, we engineered FRET pairs by fusing Halo‐tag with green fluorescent protein (GFP) and red fluorescent protein (RFP), using Halo dyes **O‐Rho** and **Si‐Rho** as acceptors (Scheme 1, right). This design yielded two chemigenetic fluorophores (GLH‐O and CLH‐Si) with enhanced SNR, even at low protein expression levels. To further optimize FRET efficiency, we systematically shortened the linker between mCherry and Halo‐tag, resulting in the development of the CH‐Si fluorophore. This system enabled no‐wash, four‐color super‐resolution imaging of target proteins and dynamic tracking of mitochondrial interactions with endoplasmic reticulum and lysosomes. Additionally, by incorporating the photostable mStayGold as a FRET donor, we created the SLH‐O fluorophore, which exhibits exceptional photostability for long‐term super‐resolution imaging. Our work exemplifies the power of combining chemical and genetic tools to address challenges in live‐cell imaging. By providing a generalizable framework for improving the SNR of fluorescent dyes, this strategy opens new avenues for real‐time, quantitative, and long‐term super‐resolution dynamic tracking of target proteins, underscoring the transformative potential of chemical biology in advancing biological research.

## 2 Results and Discussion

### 2.1 Evaluation of Halo‐Tag‐Based Dyes

**Figure 1:**
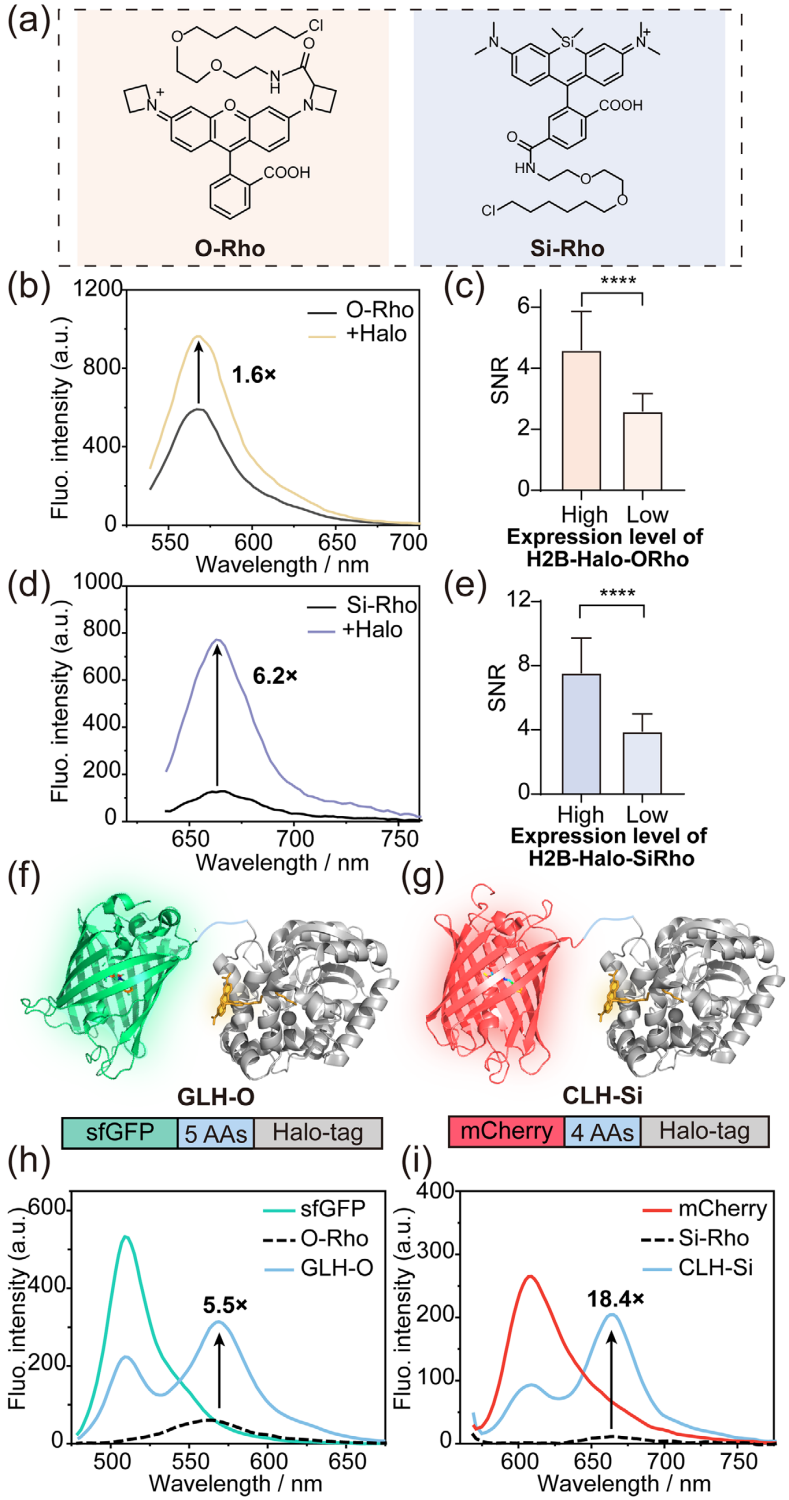
Characterization of traditional fluorogenic probes and chemigenetic FRET probes. a,c) Emission spectra of a) **O‐Rho** and c) **Si‐Rho** in the presence and absence of excess Halo‐tag. **O‐Rho**: λ_ex_ = 530 nm, **Si‐Rho**: λ_ex_: 630 nm. b,d) At the same dye labeling concentration (**O‐Rho**: 0.1 µm; **Si‐Rho**: 0.3 µm), the difference of SNR with varying H2B‐Halo protein expression levels in HeLa cells. Data are means ± S.D.; n = 40–50 cells; Each group represents three independent experiments separately; Unpaired two‐tailed Student's t‐test, ^****^ *p* ≤ 0.0001. e,f) Schematic representation of the GLH‐O and CLH‐Si structure of Halo‐tag (PDB: 6y7a) fused at the C‐terminal of sfGFP (PDB: 2B3P) and mCherry (PDB: 2H5Q). g) Fluorescence emission spectra of **O‐Rho** with and without the addition of GLH (λ_ex_: 470 nm). h) Fluorescence emission spectra of **Si‐Rho** with and without the addition of CLH (λ_ex_: 561 nm).

Rhodamine (Rho) and its derivatives are widely used for protein labeling and live‐cell imaging due to their exceptional photophysical properties, including high brightness and photostability.^[^ ^29^ ^]^ Following established synthetic protocols,^[^ ^32^, ^33^ ^]^ we prepared two Halo‐tag probes, **O‐Rho** and **Si‐Rho** (Figure S1, Supporting Information). Spectroscopic characterization in vitro revealed distinct absorption and fluorescence activation behaviors for these probes upon binding to Halo‐tag. **O‐Rho**, which predominantly exists as fluorescent zwitterions in aqueous solution, exhibited only a 1.6‐fold increase in fluorescence emission at 569 nm upon Halo‐tag binding (**Figure** **1**a; Figure S1a, Supporting Information). In contrast, **Si‐Rho**, which primarily adopts a non‐fluorescent spironolactone in aqueous solution, showed a 6.2‐fold increase in fluorescence emission at 662 nm upon binding to Halo‐tag (Figure 1c; Figure S1b, Supporting Information). These results are consistent with previous reports.^[^ ^17^ ^]^

Next, we used **O‐Rho** and **Si‐Rho** to stain living cells (Figures S2a and S3a, Supporting Information). Both dyes showed significant fluorescence enhancement in both mitochondria and lysosomes, indicating that this nonspecific binding may be affected by multiple organelle microenvironments, such as pH, polarity, and hydrophobic protein cavities,^[^ ^21^, ^22^, ^23^, ^24^ ^]^ thereby shifting the spirocyclization equilibrium from the nonfluorescent (L) form to the fluorescent (Z) form, turning on the fluorescence (Scheme 1b). As the dye concentration increased, background fluorescence also intensified (Figures S2b and S3b, Supporting Information). When HeLa cells overexpressing H2B‐Halo protein were labeled with these probes (Figures S2c and S3c, Supporting Information), the signal‐to‐noise ratio (SNR)—defined as the ratio of nuclear to cytoplasmic fluorescence—decreased significantly with higher dye concentrations (Figures S2d and S3d, Supporting Information). Notably, at the same dye concentration, cells with lower H2B‐Halo expression levels exhibited a lower SNR (Figure 1b,d). **O‐Rho** showed a higher SNR in live‐cell imaging compared to its in vitro performance, likely due to the nuclear accumulation of the dye caused by H2B‐Halo overexpression. Overall, the fluorescence turn‐on from nonspecific binding in fluorogenic dyes significantly affects the SNR in live‐cell imaging.

### 2.2 Design and Construction of Chemigenetic Fluorophores

To address the limitations of nonspecific fluorescence turn‐on, we developed a chemigenetic fluorophore strategy based on Förster resonance energy transfer (FRET). **O‐Rho** and **Si‐Rho**, with their distinct emission wavelengths and fluorescence turn‐on properties (Figure 1a,c), served as ideal candidates for demonstrating the versatility of this approach. For FRET, we considered the optimal overlap of the donor's absorption and the acceptor's emission spectra, the high quantum yield of the donor, and the strong extinction coefficient of the acceptor (Figure S4, Supporting Information).^[^ ^28^ ^]^ Specifically, we chose sfGFP and mCherry as donors for **O‐Rho** and **Si‐Rho**, respectively, and fused each FP to Halo‐tag via a flexible linker (Figure 1e,f). The fusion proteins were expressed and purified, and their correct molecular weights were verified by SDS‐PAGE. Furthermore, we utilized the Halo‐tag dye Naph‐A2‐Halo^[^ ^32^ ^]^ to demonstrate that all fusion proteins could be covalently bound to the Halo‐tag dye and effectively labeled (Figures S5 and S6, Supporting Information). This yielded two chemigenetic fluorophores: sfGFP‐linker‐Halo‐**O‐Rho** (GLH‐O) and mCherry‐linker‐Halo‐**Si‐Rho** (CLH‐Si). The linkers used in the design consisted of 4–5 amino acids (Table S1, Supporting Information), chosen to maintain the activity of both the fluorescent protein (FP) and the Halo‐tag protein while ensuring that the fluorescence properties of the respective fluorophores were not compromised. Additionally, the short linker design was crucial for ensuring an optimal FRET distance, allowing for efficient energy transfer between the fluorophores.

Spectroscopic analysis of GLH‐O revealed efficient FRET, with **O‐Rho** fluorescence (λ_em_ = 565 nm) turning on upon excitation of sfGFP (λ_ex_ = 470 nm). The decrease in donor intensity and concomitant increase in acceptor fluorescence confirmed FRET, with an efficiency of 58.1% (Figure 1g). Similarly, CLH‐Si exhibited a FRET efficiency of 64.9% (Figure 1h). In contrast, free **O‐Rho** and **Si‐Rho** in solution showed minimal fluorescence at the same excitation wavelengths, resulting in 5.5‐fold and 18.4‐fold increases in fluorescence at the acceptor emission wavelengths for GLH‐O and CLH‐Si, respectively.

These enhancements far exceeded the fluorescence turn‐on ratios observed for **O‐Rho** (1.6‐fold) and **Si‐Rho** (6.2‐fold) alone (Figure 1a,c), demonstrating the significant improvement in fluorescence intensity achieved by our chemigenetic fluorophore strategy.

### 2.3 Wash‐Free Live‐Cell Imaging of Chemigenetic Fluorophore

To evaluate the performance of our chemigenetic fluorophores in live‐cell imaging, we first assessed the FRET effect of GLH‐O and CLH‐Si in HeLa cells. Cells were transiently transfected with fusion proteins H2B‐GLH and H2B‐CLH (H2B: human histone), and fluorescence imaging was performed before and after dye labeling. Under 488 nm excitation of the sfGFP donor, GLH‐O exhibited minimal fluorescence in the FRET emission channel (580–653 nm) prior to **O‐Rho** labeling (Figure S7a, Supporting Information). Upon addition of **O‐Rho**, fluorescence in the donor channel (500–550 nm) decreased significantly, while the FRET channel signal increased, confirming efficient FRET in GLH‐O. Similarly, FRET was observed in HeLa cells expressing H2B‐CLH‐Si under 561 nm excitation (emission collected at 663–703 nm, Figure S7b, Supporting Information), consistent with our in vitro spectroscopic results.

**Figure 2:**
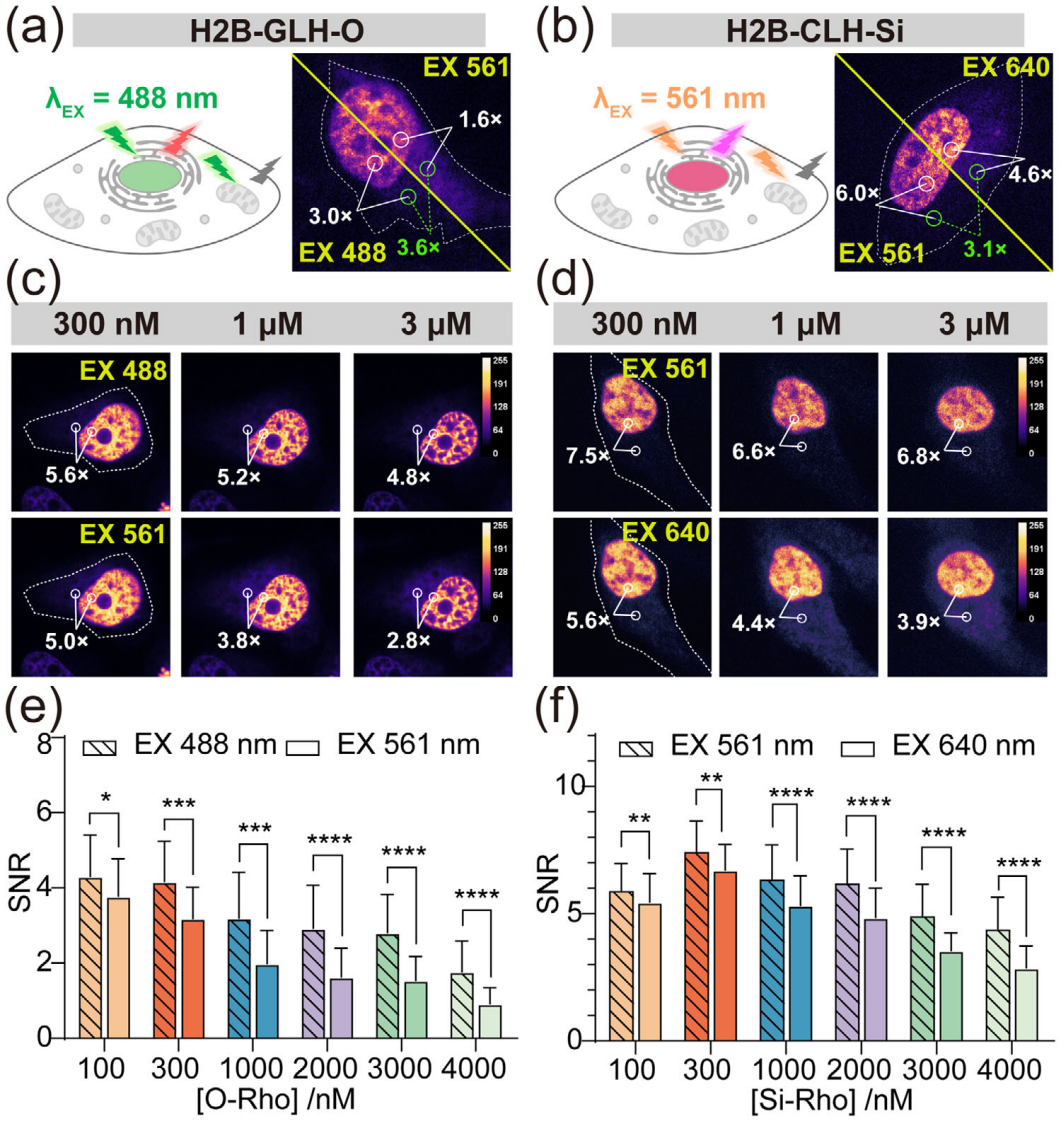
Wash‐free live‐cell imaging of chemigenetic fluorophores. a,b) schematic diagram and the wash‐free confocal imaging of chemigenetic fluorophores GLH‐O and CLH‐Si [**O‐Rho**]: 2 µm; [**Si‐Rho**]: 2 µm. c,d) Comparison of no‐wash imaging in the FRET channel and the acceptor channel for c) H2B‐GLH‐O or d) H2B‐CLH‐Si protein labeled with varying concentrations of Rho. Calibration bar shows the normalized fluorescence intensity. e,f) Statistical analysis of the SNR in the FRET channel and Rho channel for HeLa cells expressing e) GLH‐O or f) CLH‐Si under different dye concentration treatments. Data are means ± S.D.; n = 40–50 cells; Each group represents 3 independent experiments separately; Unpaired two‐tailed Student's t‐test, ^*^ *p* ≤ 0.05, ^**^ *p* ≤ 0.01, ^***^ *p* ≤ 0.001, ^****^ *p* ≤ 0.0001.

The genetically encoded fluorescent proteins (FPs) serve as energy donors for the Rho dyes, effectively blue‐shifting the excitation wavelength of the target‐bound dyes. This design ensures that only target‐bound dyes are excited, while nonspecifically bound dyes remain dark, thereby minimizing background fluorescence (**Figure** **2**a,b). To validate the wash‐free imaging capability of our fluorophores, we performed imaging at low protein expression levels. HeLa cells expressing H2B‐GLH and H2B‐CLH were incubated with 2 µm **O‐Rho** and **Si‐Rho**, respectively, and imaged without washing. Single‐cell imaging revealed that background fluorescence under short‐wavelength excitation (GLH‐O: λ_ex_ = 488 nm; CLH‐Si: λ_ex_ = 561 nm) was significantly lower than under conventional dye excitation wavelengths (GLH‐O: λ_ex_ = 561 nm; CLH‐Si: λ_ex_ = 640 nm). Specifically, background fluorescence was reduced by 3.6‐fold for GLH‐O and 3.1‐fold for CLH‐Si, leading to a marked improvement in SNR (Figure 2a,b). However, short‐wavelength may also excite residual non‐specific fluorescence due to some crosstalk between the FRET pairs, so the background fluorescence has not been eliminated.

To further quantify the performance of our fluorophores, we compared the SNR in the same cells after incubation with varying concentrations of **O‐Rho** and **Si‐Rho** (0.1–4 µM). The results demonstrated that the SNR in the FRET channel was consistently higher than in the dye channel across all dye concentrations (Figure 2c–f). Moreover, as dye concentration increased, the SNR improved more significantly, with GLH‐O showing a greater enhancement compared to CLH‐Si (Figure S8, Supporting Information). However, due to limitations in FRET efficiency and crosstalk between the FRET pairs, the overall SNR improvement was constrained to a 1.5‐ to 2‐fold increase. Although this improvement in SNR may seem modest, the true strength of our strategy lies in its broader applicability. It is not constrained by reaction time or complex molecular modifications and can work with any dye‐protein pair, even in cells with low expression levels of the target protein. Furthermore, in theory, the use of perfectly matched FRET pairs with minimal crosstalk can completely eliminate nonspecific background signals, thereby achieving the ideal genetically encoded fluorescence system.

### 2.4 The Improvement of FRET Efficiency further Enhanced the SNR of Chemigenetic Fluorophores

**Figure 3:**
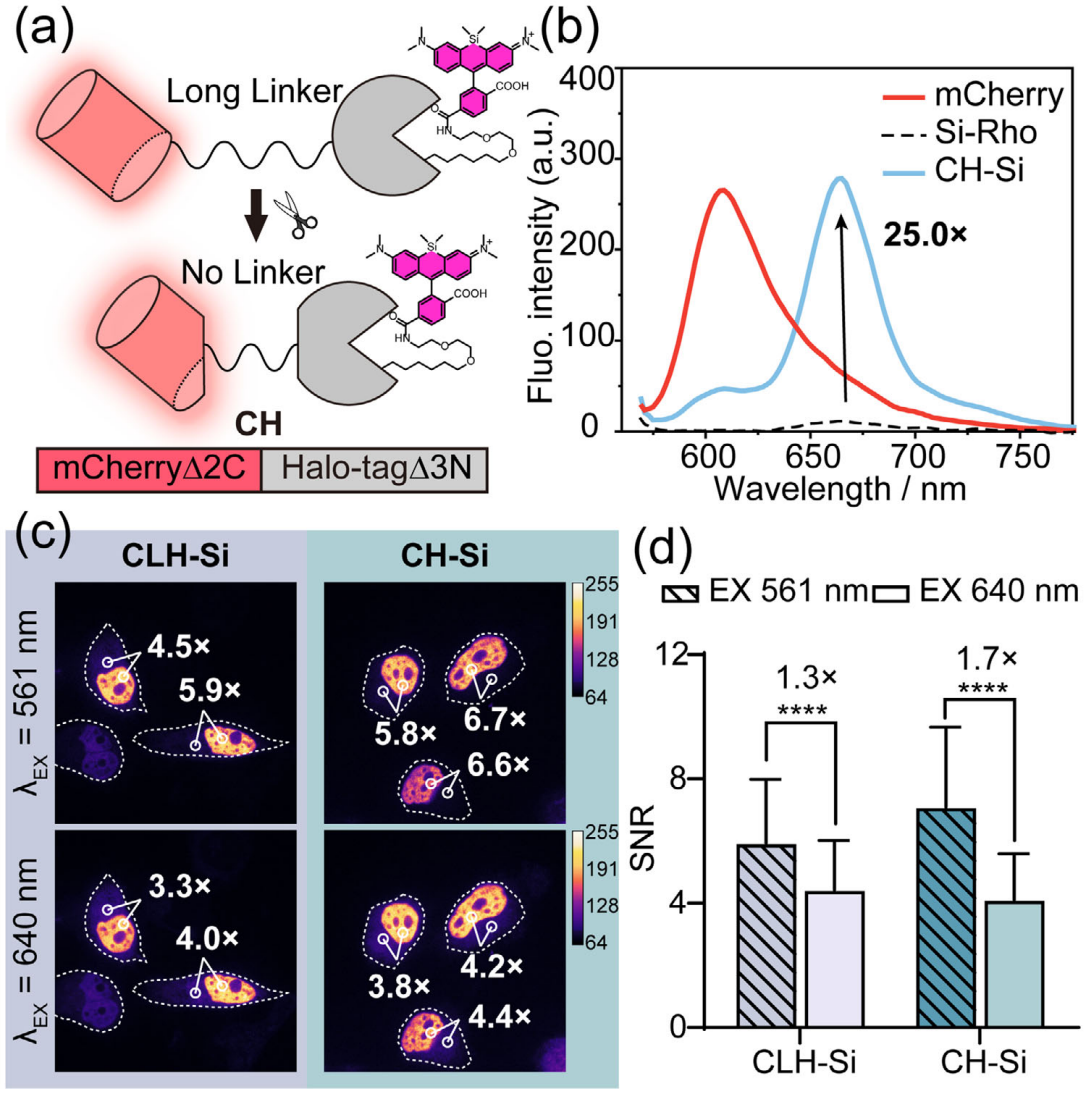
Development of chemigenetic fluorophores with higher SNR. a) Protein engineering schematic of CH fluorogenic probe. b) Fluorescence emission spectra of CH protein with (blue line) and without (red line) the addition of Si‐Rho (λ_ex_: 561 nm). The numbers indicate the ratio between the fluorescence of CH‐Si and free Si‐Rho (black dot line) at 662 nm (λ_ex_: 561 nm). c) Comparison of no‐wash imaging in the FRET channel and the Si‐Rho channel for H2B‐CLH‐Si or H2B‐CH‐Si. [Si‐Rho]: 2 µm. Calibration bar shows the normalized fluorescence intensity. d) Statistical analysis of the SNR in the FRET channel and Rho channel from the images of (c). Data are means ± S.D.; n = 40–50 cells; Each group represents three independent experiments separately; Unpaired two‐tailed Student's t‐test, ^****^ *p* ≤ 0.0001.

The CLH‐Si probe, with its near‐infrared emission and high SNR, holds significant potential for biological imaging in complex systems. To further optimize its performance, we focused on improving the FRET efficiency of CLH‐Si. While fluorescence crosstalk in the mCherry‐SiRho FRET pair inevitably contributes to background signal, increasing FRET efficiency can enhance the brightness of the chemigenetic fluorophore, thereby improving its SNR. We truncated the linker amino acids between mCherry and Halo‐tag fusion protein to obtain mCherry‐Halo (hereinafter referred to as CH, **Figure** **3**a). The CH protein was expressed, purified, and labeled with **Si‐Rho** to create the CH‐Si fluorophore (Figure S6, Supporting Information). Spectroscopic characterization revealed a significant enhancement in energy transfer efficiency, with FRET efficiency increasing from 64.9% for CLH‐Si to 83.7% for CH‐Si (Figure 3b). Under 561 nm excitation, CH‐Si exhibited a 25‐fold increase in fluorescence at the dye emission wavelength (662 nm) compared to free **Si‐Rho** in solution, demonstrating the dramatic improvement in brightness achieved through FRET optimization.

Furthermore, HeLa cells were transiently transfected with CH‐H2B and labeled with 2 µm **Si‐Rho** for no‐wash imaging after 12 h of expression. The SNR of fluorescence imaging in the same cell at 561 nm excitation was improved compared to 640 nm excitation (Figure 3c). When compared to CLH‐Si, the SNR of the genetically encoded fluorescence channel increased from 4.87 ± 1.68 to 5.92 ± 1.32, while the SNR of the dye channel remained largely unchanged (CLH‐Si: 3.59 ± 0.792; CH‐Si: 3.90 ± 0.483). This resulted in an overall SNR improvement from 1.3‐fold to 1.7‐fold (Figure 3d). As expected, CH‐Si exhibited a higher SNR, confirming the effectiveness of the FRET optimization.

### 2.5 The Universality of CH‐Si for Labeling Different Proteins in Living Cells

In previous experiments, the CH‐Si probe successfully labeled the H2B protein in HeLa cells (Figure S8a, Supporting Information). Expanding on this, we continued to fuse CH to the N terminus of various proteins, including the mitochondrial outer membrane protein TOMM20, the endoplasmic reticulum tail‐anchored protein Sec61β, and the lysosome‐targeting GTPase Rab7. After 12 h of transient transfection, we performed no‐wash fluorescence imaging. The imaging results revealed that CH‐Si efficiently labeled different cellular organelles, with no interference in the correct localization of these subcellular proteins (Figure S9b–d, Supporting Information). Furthermore, CH‐Si exhibited good SNR in imaging different organelles (Figure S9e, Supporting Information). We further confirmed the correct subcellular labeling and precise localization of these fusion proteins by co‐staining with commercially available organelle‐specific dyes during confocal fluorescence imaging (Figure S10, Supporting Information), ensuring that the use of CH‐Si did not interfere with the functionality of the target proteins. These results demonstrate the broad applicability of CH‐Si as a versatile tool for biological labeling in living cells.

### 2.6 Using CH‐Si for No‐Wash Four‐Color SIM Imaging

The study of most biological functions requires the simultaneous observation of multiple biomolecules and their interactions.^[^ ^34^ ^]^ To achieve this, fluorescent probes for multicolor live‐cell imaging must have not only sufficient spectral resolution to distinguish various fluorescently labeled structures, but also characteristics suitable for no‐wash imaging to reduce the complexity of sample handling and improve imaging efficiency.^[^ ^35^, ^36^ ^]^ The blue‐shifted excitation of CH‐Si provides an extended Stokes shift characteristic (75 nm), making it compatible with fluorophores of conventional channels for multichannel imaging. Here, we first examined the feasibility of dual‐labeling using CH‐Si and traditional RFPs for confocal imaging. The critical requirement for this application was that CH‐Si exhibits minimal residual fluorescence in the donor emission channel. To assess this, we labeled H2B protein in HeLa cells with CH‐Si and mCherry, respectively, and quantified the fluorescent signals from donor channel (λ_ex_ = 561 nm, collection 580–653 nm) and FRET channel (λ_ex_ = 561 nm, collection 663–703 nm). By calculating the fluorescence ratio of the two channels, we determined the residual fluorescence level of CH‐Si in the donor channel (Figure S11a, Supporting Information). The results showed that the fluorescence ratio of CH‐Si was significantly reduced compared with mCherry. This indicates that CH‐Si has lower fluorescence in the donor channel and stronger FRET emission, which could be easily distinguished from traditional RFPs. Figure S11b (Supporting Information) showed dual‐color imaging in HeLa cells using mCherry and CH‐Si, where lysosomes (green channel) and mitochondria (red channel) subcellular organelles can be distinguished by different colors without obvious fluorescence interference.

**Figure 4:**
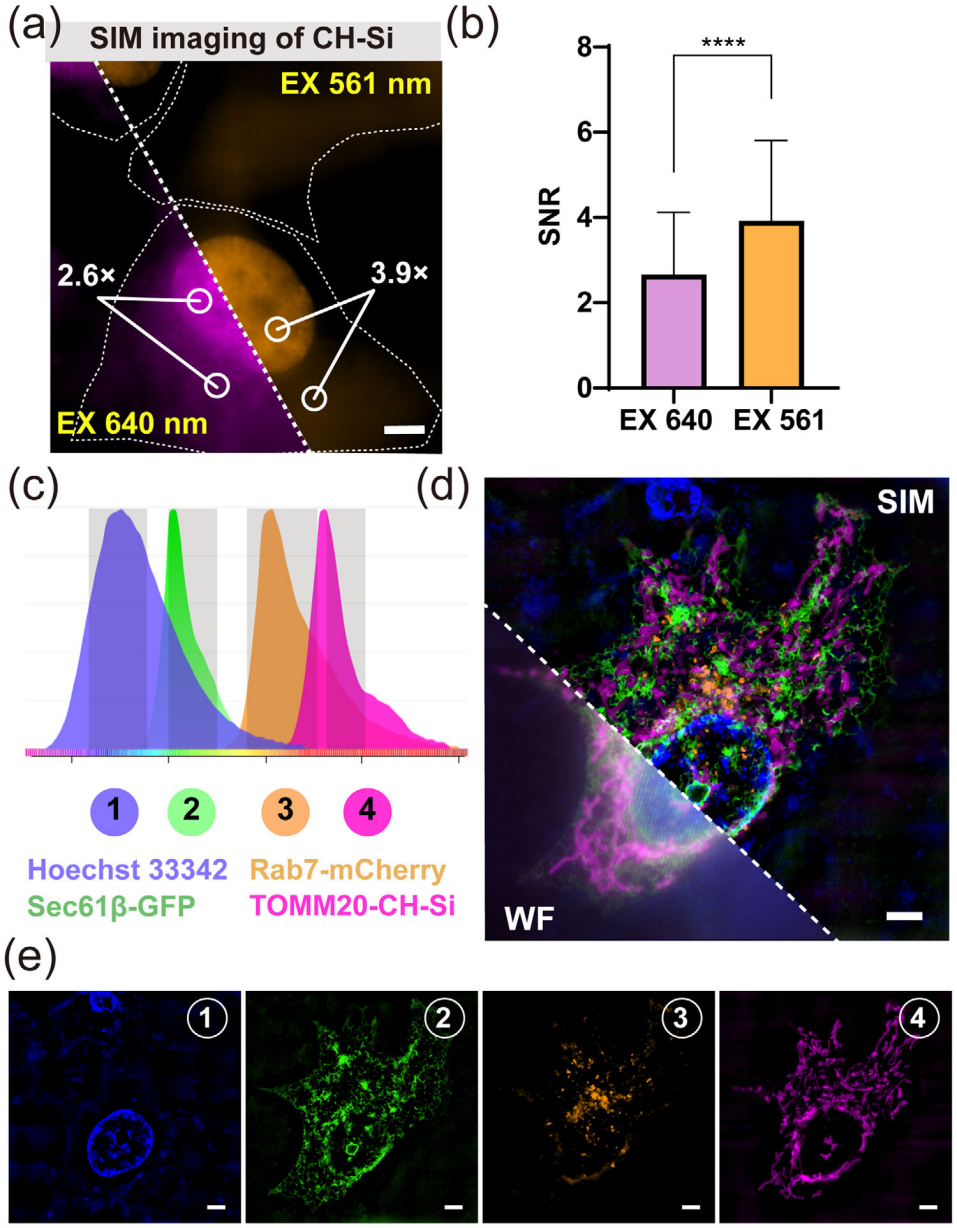
CH‐Si for wash‐free live‐cell SIM imaging. a) Comparison of no‐wash SIM imaging in the FRET channel and the Si‐Rho channel for H2B‐CH‐Si. [Si‐Rho]: 2 µm. Scale bar: 5 µm. b) Statistical analysis of the SNR in the FRET channel and Rho channel from the images of (a). Data are means ± S.D.; n = 40–50 cells; Each group represents 3 independent experiments separately; Unpaired two‐tailed Student's t‐test, ****p ≤ 0.0001. c) Emission spectra of the different fluorophores used for four‐color imaging. The grey area shows the collection wavelength range from the different channels, which marked with different color numbers. d) Four‐color confocal and SIM images of nucleus stained with Hoechst 33 342 (blue ①), Sec61β labeled with EGFP (green ②), Rab7 labeled with mCherry (orange ③), and TOMM20 labeled with CH‐Si (pink ④). e) The bottom panels show the separated channel images corresponding to the overlay in (b). Scale bar: 5 µm.

Structured illumination microscopy (SIM) is a powerful imaging technique that can provide high‐resolution images of biological structures in living cells.^[^ ^37^ ^]^ Similar to traditional microscopy, the application effect of SIM in living‐cell is largely limited by the types and number of available fluorescent probes, which directly affecting its performance and application in multi‐color imaging.^[^ ^38^ ^]^ First, we compared the SNR of CH‐Si (λ_ex_ = 561 nm, λ_em_ = 700 ± 75/2 nm) and Halo‐Si (λ_ex_ = 640 nm, λ_em_ = 663–738 nm), showing that CH‐Si also has a better SNR in SIM super‐resolution imaging (**Figure** **4**a,b). Then we combined CH‐Si with several traditional fluorophores for four‐color imaging, where CH‐Si was labeled on TOMM20, Hoechst 33 342 was stained on the nucleus, EGFP was fused to Sec61β, and mCherry was labeled on Rab7 (Figure 4c). By using these four different fluorophores, we successfully achieved SIM imaging of four substructures (Figure 4d,e): the nucleus (blue ①), endoplasmic reticulum (green ②), lysosome (orange ③), and mitochondria (pink ④). These results suggested that the combination of CH‐Si with other fluorescent probes provided a powerful tool for monitoring the dynamics of multiple organelles.

### 2.7 SIM Dynamic Imaging of Mitochondria with CH‐Si

The dynamic behavior of various biomolecules and organelles in cells is often extremely complex and rapidly changing. Precise positioning and high‐resolution imaging are essential for accurately capturing these dynamic processes.^[^ ^39^ ^]^ The position and movement of mitochondria in living‐cells play a key role in cellular energy metabolism, signal transduction, and apoptosis.^[^ ^40^, ^41^ ^]^ Mitochondria carry out their functions through a close network of interactions with other organelles such as the endoplasmic reticulum (ER) and lysosomes.^[^ ^42^, ^43^ ^]^ we utilized CH‐Si‐based SIM imaging to track the dynamics interactions of mitochondria with the ER and lysosomes.

**Figure 5:**
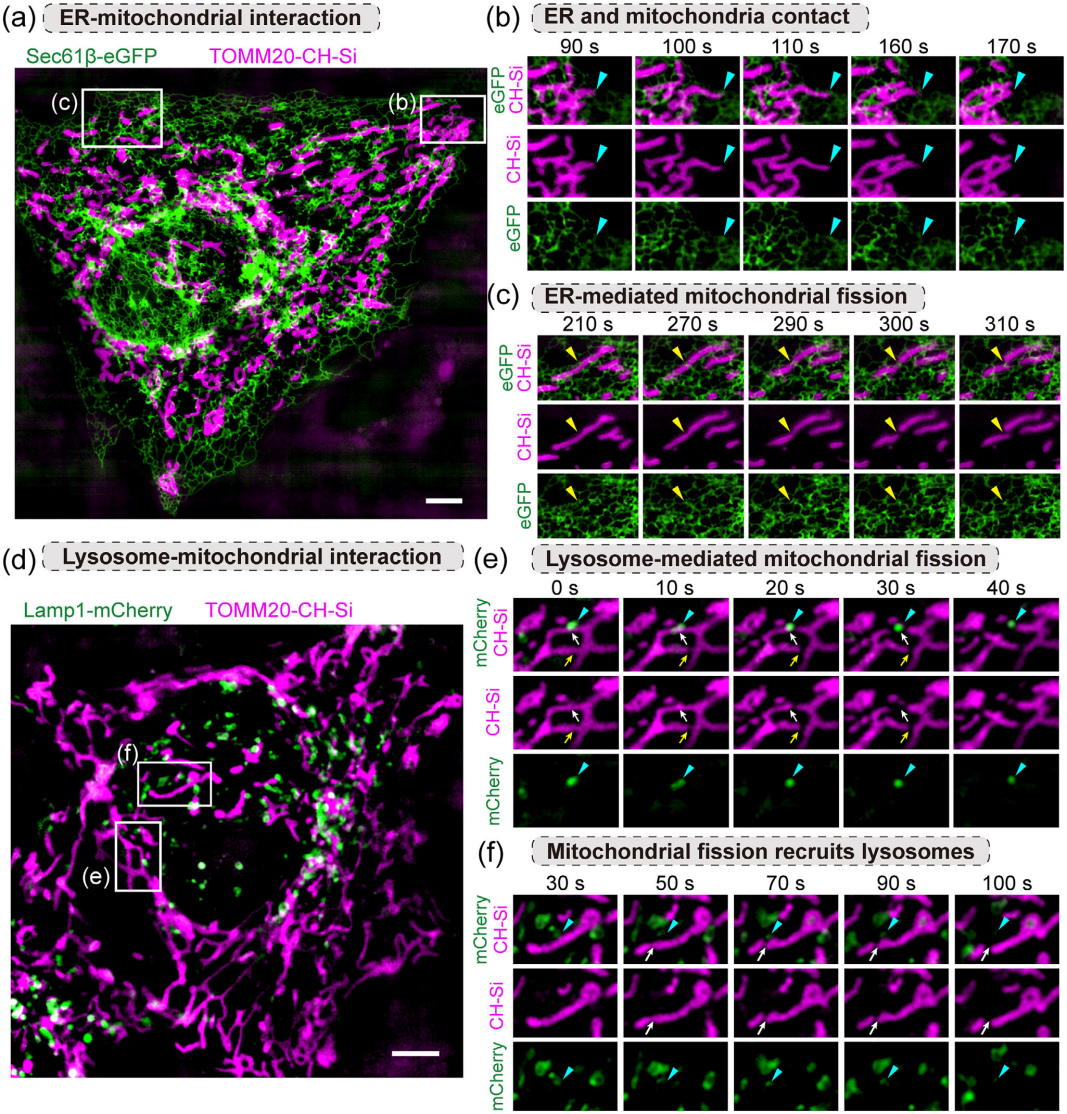
Dual‐color SIM imaging of mitochondria dynamic using CH‐Si. a) Dual‐color SIM image of HeLa cells co‐expressing GFP‐Sec61β and TOMM20‐CH‐Si. The endoplasmic reticulum and mitochondria are represented in green and pink channels, respectively. Scale bar: 5 µm. b) Magnified images of the boxed region in (a). The blue arrows indicate the contact event between mitochondria and the endoplasmic reticulum. c) Magnified images of the boxed region in (a). The yellow arrow indicates the location where endoplasmic reticulum‐induced mitochondrial fission event occurred. d) Dual‐color SIM image of HeLa cells co‐expressing mCherry‐Rab7 and TOMM20‐CH‐Si. The lysosome and mitochondria are represented in green and pink channels, respectively. Scale bar: 5 µm. e,f) Magnified images of the boxed region in (d). The two groups of images represent the two modes of action of lysosomes in the fission of mitochondria.

To observe the interaction between mitochondria and the ER, we co‐expressed TOMM20 protein labeled with CH‐Si and EGFP‐Sec61β in HeLa cells and performed dual‐color SIM imaging. This enabled continuous monitoring of the mitochondrial‐ER interactions over 20 min (**Figure** **5**a–c). The boxes in Figure 5 highlight the details of the interaction between mitochondria and ER at different locations. In Figure 5b, we observed a significant morphological change in mitochondrion at 100 s, indicated by the blue arrow, where it elongated and briefly contacted the adjacent ER. Over time (110‐160 s), the mitochondrial morphology gradually recovered, shrank and broke away from the contact with ER. The interaction between mitochondria and ER is not limited to physical contact, but also has an important impact on the function and morphology of mitochondria. In another observation (Figure 5c), we tracked a mitochondrial fission event (300‐310 s) after a rapid interaction with the ER (270 s). The combined labeling of CH‐Si and EGFP can reveal the complex and delicate dynamic relationship between mitochondria and endoplasmic reticulum.

Next, we examined the dynamic interaction between mitochondria and lysosomes by labeling lysosomal Rab7 protein with mCherry. We successfully demonstrated the high‐resolution separation of the two fluorescence signals, ensuring the resolution accuracy of mitochondria and lysosomes (Figure 5d–f; Figure S9b, Supporting Information). In Figure 5e, we observed the dynamic process of lysosomes participating in mitochondrial fission. The lysosome indicated by the blue arrow induced the fission of the mitochondria (30 s) after contacting the mitochondria (10 s). This indicates that the lysosomes play a significant role in the process of mitochondrial fission.^[^ ^44^ ^]^ However, in the adjacent key region indicated by the yellow arrows, mitochondria did not interact with lysosomes during the observation period, suggesting that lysosomes are not always required for mitochondrial fission and that there may be different patterns of mitochondrial fission. In addition, we also observed another interaction pattern between mitochondria and lysosomes. In Figure 5f, after mitochondria (white arrow) underwent fission (50‐70 s), the lysosomes (blue arrow) moved quickly to the fission site at 70 s and remained there for some time. This suggests that lysosomes may carry out important molecular transport or other biological processes.^[^ ^45^ ^]^ Subsequently, the lysosomes moved out of the fission site at 90 s. This phenomenon may indicate that lysosomes perform certain functional tasks on post‐fission mitochondrial, such as membrane repair, material exchange, or signal transduction.^[^ ^46^ ^]^

### 2.8 Chemigenetic Fluorophores with High Photostability

**Figure 6:**
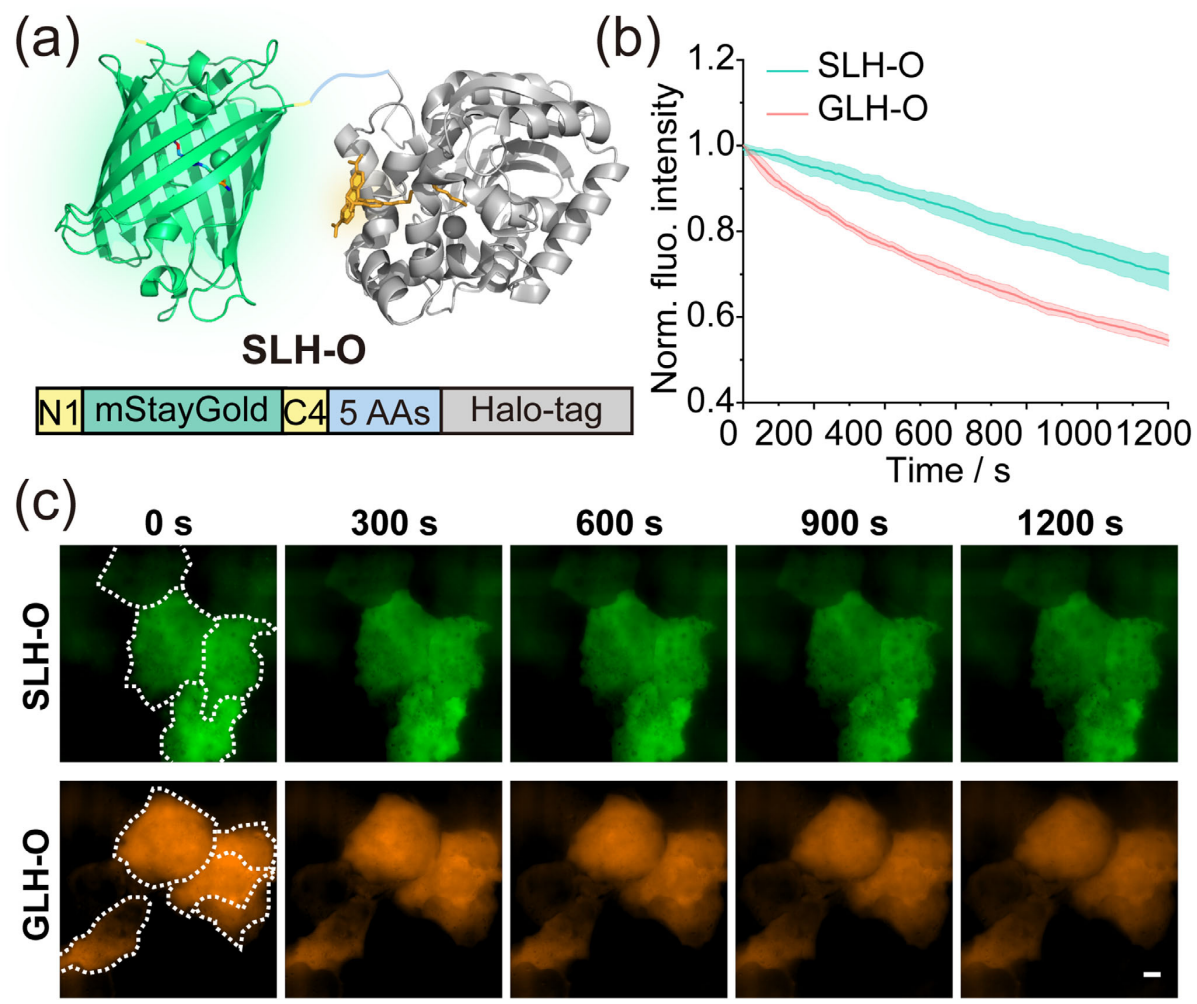
Construction and characterization of photostable chemigenetic fluorophores. a) Schematic representation of the structure of Halo‐tag (PDB: 6y7a) fused at the C‐terminal of mStayGold (PDB: 8BXT). b) Photobleaching dynamics comparison of SLH‐O and GLH‐O with SIM super‐resolution imaging. Each curve represents the average of three independent experiments. n > 10 cells. Laser export power: 488 nm, 0.16 kW cm^−2^. c) SIM photobleaching images over time of SLH‐O and GLH‐O expressed in the cytoplasm.

To achieve long‐term super‐resolution imaging, it is crucial to develop chemigenetic fluorophores with excellent photostability. However, traditional fluorescent proteins often exhibit limited photostability, and their combination with small molecule dyes can further compromise dye stability. To address this challenge, we selected the recently developed highly photostable monomeric green fluorescent protein mStayGold,^[^ ^47^ ^]^ and fused it with the Halo‐tag. In conjunction with the **O‐Rho** dye (**Figure** **6**a; Figure S12, Supporting Information), we constructed a new chemigenetic fluorophore mStayGold‐Linker‐Halo‐**ORho** (hereinafter referred to as SLH‐O). To assess the photostability of SLH‐O, we conducted long‐term SIM super‐resolution imaging. The results demonstrated that SLH‐O exhibited significantly enhanced photostability compared to GLH‐O (Figure 6b,c). SLH‐O also showed excellent resistance to photobleaching under strong laser irradiation (488 nm, 0.16 kW cm^−2^). This finding proves that the combination of mStayGold with **O‐Rho** dye can significantly enhance the photostability of chemigenetic fluorophores, providing a valuable tool for long‐term dynamic tracking and no‐wash labeling in complex biological systems.

## 3 Conclusion

In this paper, we successfully developed a universal strategy to convert exogenous fluorescent dyes into probes with genetically encoded fluorescence, which significantly improved the SNR of fluorescent dyes in no‐wash super‐resolution imaging of living cells. We validated the effectiveness of this strategy by applying chemigenetic fluorophores (GLH‐O, CLH‐Si) in HeLa cells, and found that the SNR in FRET channel was higher than that in the dye channel under all **O‐Rho**/**Si‐Rho** dye concentrations (0.1 µM‐4 µM). Additionally, the improved FRET efficiency (83.7%) in the CH‐Si system effectively reduces background fluorescence, providing clearer and more accurate imaging of subcellular structures, and improving the imaging SNR from 1.3‐fold to 1.7‐fold. In addition, we successfully combined CH‐Si with other fluorophores to achieve four‐color SIM imaging and demonstrated the dynamic interactions between mitochondria and the endoplasmic reticulum/lysosomes through two‐color no‐wash imaging. Furthermore, by introducing the SLH construct, we achieved long‐term super‐resolution imaging stability.

Despite these advancements, challenges remain. The SNR enhancement of CH‐Si and GLH‐O is currently limited to approximately 1.5‐ to 2‐fold due to fluorescence bleed‐through. With an ideal FRET pair and high FRET efficiency, this strategy allows any fluorophores to maintain high signal stability and imaging quality during protein labeling and tracking, and is not limited by low protein expression environments, providing a reliable tool for long‐term super‐resolution tracking of complex cell dynamic processes.

## Conflict of Interest

The authors declare no conflict of interest.

## Author Contributions

Concepts were conceived by Z.X.; L.M. and X.Z. designed the experiments and analyzed the data; X.Z. performed in vitro experiments; X.Z., C.Y., J.S., J. Z. and G.W. performed live cells experiments; N.X. and Q.Q. performed compounds synthesis; L.M., X.Z., and Z.X. wrote the manuscript.

## Supporting information



Supporting Information

## Data Availability

The data that support the findings of this study are available from the corresponding author upon reasonable request.
